# Automatic Recognition and Classification System of Thyroid Nodules in CT Images Based on CNN

**DOI:** 10.1155/2021/5540186

**Published:** 2021-05-27

**Authors:** Wenjun Li, Siyi Cheng, Kai Qian, Keqiang Yue, Hao Liu

**Affiliations:** ^1^Key Laboratory of RF Circuits and Systems, Ministry of Education, Hangzhou Dianzi University, Hangzhou, Zhejiang, China; ^2^Department of Radiology, The No. 1 People's Hospital of Pinghu, Jiaxing, Zhejiang, China

## Abstract

Thyroid nodule lesions are one of the most common lesions of the thyroid; the incidence rate has been the highest in the past thirty years. X-ray computed tomography (CT) plays an increasingly important role in the diagnosis of thyroid diseases. Nonetheless, as a result of the artifact and high complexity of thyroid CT image, the traditional machine learning method cannot be applied to CT image processing. In this paper, an end-to-end thyroid nodule automatic recognition and classification system is designed based on CNN. An improved Eff-Unet segmentation network is used to segment thyroid nodules as ROI. The image processing algorithm optimizes the ROI region and divides the nodules. A low-level and high-level feature fusion classification network CNN-F is proposed to classify the benign and malignant nodules. After each module is connected in series with the algorithm, the automatic classification of each nodule can be realized. Experimental results demonstrate that the proposed end-to-end thyroid nodule automatic recognition and classification system has excellent performance in diagnosing thyroid diseases. In the test set, the segmentation IOU reaches 0.855, and the classification output accuracy reaches 85.92%.

## 1. Introduction

Thyroid nodule lesions are the most common lesions of the thyroid, and the overall human prevalence can reach 19% to 46% [1]. Lesions can be divided into benign and malignant. Compared with benign nodules, malignant nodules have an irreversible impact on human health. The necessary condition for clinically correct treatment of malignant nodules is to accurately detect benign and malignant thyroid nodules [2].

In order to better solve this problem, many scholars have done a lot of research using computer-aided diagnosis technology. Nugroho used adaptive median filter and bilateral filtering for preprocessing the ultrasound images, eight geometric features were used to support vector machine (SVM) for classification, and the accuracy of thyroid nodule lesions is 92.30% [3]. Wang proposed a deep learning method to diagnose thyroid nodules using multiple ultrasound images in an examination as input. An attention-based feature aggregation network is proposed to automatically integrate the features extracted from multiple images in one examination, utilizing different views of the nodules to improve the performance of recognizing malignant nodules in the ultrasound images. In the self-built large database, the classification of benign and malignant can reach 87.32% [4]. Iakovidis et al. proposed encoding the ultrasonic features by antinoise representation and used the fusion of fuzzy local binary pattern and fuzzy gray histogram features to classify the ultrasonic features by polynomial kernel support vector machine. According to the area under the ROC curve, the classification accuracy reached 97.5% [5].

The above researches are based on ultrasound images and have achieved good results, but compared with CT images, ultrasound images have some problems, such as low definition, less useful information, and so on [6], and there is a big difference between the two images, which cannot achieve the simple moving of the method.

Now, in the study of thyroid nodules based on CT, Peng used the first-order texture features in nonenhanced CT images and support vector machine analysis to classify the nodules, and the accuracy reached 88% [7]. Liu used traditional machine learning methods, the recognition performance of thyroid nodules was evaluated according to 17 first-order statistics and gray cooccurrence matrix features. The sensitivity, specificity, positive predictive value, and negative predictive value of the final algorithm were 0.8673, 0.9105, 0.9130, 0.8269, 0.8235, and 0.9146, respectively [8]. Although the above researches have reached a high level in the study of thyroid classification, they require the prerequisite for the nodule information to be additionally labeled, and it is impossible to achieve high-efficiency data processing. On this basis, Zhao et al. designed an improved Unet architecture, Dense-Unet, to achieve the region of interest (ROI) segmentation of thyroid nodules and used multidimensional input fusion CNN model to achieve the classification of benign and malignant nodules. However, limited by the algorithm design, the algorithm is only suitable for single-nodule images, which cannot meet the situation of single picture and multiple nodule [9].

In order to solve the above problems, this study collected and established a thyroid nodule enhanced CT image database, aiming to realize the integration of thyroid nodule recognition and classification diagnosis. In CT images, the distinction between benign and malignant thyroid mainly lies in whether the shape and edges are divergent, and there is no obvious difference in color. As a result, a single multiclass segmentation network cannot achieve high accuracy and the classification effect of benign and malignant is poor. Therefore, in this research, a CNN-based semantic segmentation network plus an image classification network is designed, and image processing algorithms are used to make the two connected in a reasonable manner, so that the segmentation network focuses on the segmentation of the thyroid nodules and the background. The classification network focuses on extracting image features of thyroid nodules to achieve benign and malignant classification. In addition, for multinodule CT images, nodules are segmented to achieve separate classification of each nodule.

The main contributions of this work are as follows:

1. Based on the traditional Unet, an improved network called Eff-Unet is proposed for the segmentation of contrast-enhanced thyroid CT images, and an improved loss function is proposed to improve the semantic segmentation performance.
2. A new thyroid nodule classification method is designed by fusing two different CNN networks.
3. The image processing algorithm is designed to adapt the output image of segmentation network to the input of classification network and realize the intelligent segmentation of multinodule CT image. The end-to-end diagnosis and classification of each nodule is realized without any additional marking tasks.

## 2. Materials and Methods

**Figure 1:**
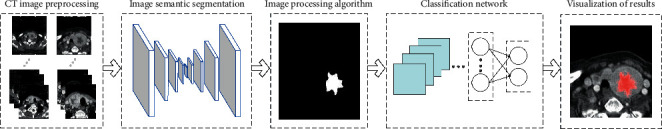
The algorithm flowchart of system. The details are as follows. (1) CT image preprocessing: data preprocessing and data enhancement. (2) Image semantic segmentation: automatic recognition and segmentation of thyroid nodules. (3) Image processing algorithm: realizing the smooth connection from the segmentation network to the classification network. (4) Classification network: achieving accurate classification of benign and malignant nodules. (5) Visualization of results: the results are mapped to the original image to facilitate observation and comparison.

The algorithm flowchart proposed in this paper is shown in Figure 1. It can be divided into 4 parts: image preprocessing part, segmentation network semantic segmentation part, image processing algorithm part, and classification network prediction and output visualization part. Input the CT image after image preprocessing into the system, realize the nodule splitting of multinodule pictures through image processing algorithms, and smoothly transition the output pictures of the segmentation network to the input of the classification network to realize end-to-end automatic recognition and integration diagnose thyroid nodules.

### 2.1. Database Characteristics

In this experiment, the CT image database of the First People's Hospital of Pinghu City and the First People's Hospital of Jiaxing City was used to construct the database. A total of 248 patients' CT images from 2013 to 2020 were obtained. All images were taken by a 16-slice spiral CT scanner (Siemens Emotion 16, one of the most popular CT scanners in the world). The basic settings were CT slice thickness of 4 nm, window width and window level of 500 and 60, and convolution kernel parameter of b50s. All the images were enhanced CT images after injection of water-soluble iodine contrast agent and scanning, which can increase the density difference between the lesion tissue and adjacent normal tissue, so as to improve the display rate of lesions [10]. All the included lesions were confirmed by biopsy or surgical pathology, and the lesion scope was clear. Because there were multiple nodules, CT images with malignant nodules were classified as malignant images in this experiment. After being selected by professional doctors, a total of 832 CT images were obtained. The detailed data of CT images data collected are shown in Table 1. The detailed data of thyroid nodes data collected are shown in Table 2.

### 2.2. Image Preprocessing

The format of CT exported image is JPEG, and the size is 512 ∗ 512 pixels. In order to avoid learning error noise, such as bones and blood vessels, the central part of CT image is intercepted uniformly, and the size of saved core area is 256 ∗ 256 pixels. The image labeling work is assisted by two professional doctors, and the LabelMe labeling tool is used to generate the mask image. In the original mask image, the pixels of the generated background area are 0, the pixels of the benign thyroid area are 1, and the pixels of the malignant thyroid area are 2. The total number of images is relatively small, which cannot meet the condition of deep learning algorithm training with a large number of data. Therefore, data enhancement is used to generate new images to improve the performance and robustness of the model. Through the data enhancement tool of Keras framework, setting appropriate rotation angle limit and offset amplitude limit, and flipping horizontally to realize various transformations of the original image, a new image is generated and input into the algorithm to trick the model into thinking that it is receiving new data, thereby making the model more powerful in processing new types of images [11].

### 2.3. Image Segmentation Model

In the automatic recognition of thyroid nodules, the Unet [12] network model is used for image semantic segmentation, and the deep learning algorithm is used to automatically divide the location, shape, and size of the nodules. The organ structure of thyroid CT image is fixed and the semantic information is not particularly rich. The fusion of different scale features is an important means to improve the segmentation performance. Low-level features have higher resolution and contain more location and detail information. However, due to less convolution, they have lower semantics and more noise. High-level features have stronger semantic information, but the resolution is very low, and the perception of details is poor. Therefore, both high-level semantic information and low-level features are very important. Consequently, the jump connection structure and U-shaped structure network of Unet can combine the high-resolution local information and the lower-resolution and larger-area information at the same time, making the segmentation of thyroid nodules more accurate [13].

**Figure 2:**
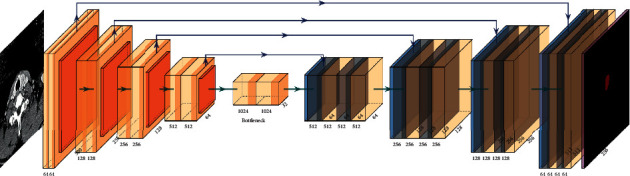
The structure of Eff-Unet.

In addition, the EfficientNet [14] network structure is selected as its backbone [15]. EfficientNet uses the depth, width, and resolution adjustment technology. Compared with other mature networks, EfficientNet has the huge advantage of number of parameters and computation [16]. It can extract image features quickly and efficiently, which lays the foundation for the realization of accurate segmentation of Unet network. The Eff-Unet designed in this experiment is shown in Figure 2. As shown in Figure 2, its basic structure is similar to the Unet network, but the downsampling encoder part rewrites its original Vgg-like structure and chooses the EfficientNet with excellent performance.

For the segmentation network, the main function is to automatically label the nodule part, and there is no need to distinguish between benign and malignant nodules. Therefore, the mask image needs additional processing before it is input into the network. When there is a malignant area with a pixel value of 2, it is automatically converted to a pixel value of 1. The output image and input image of the model are the same size. After specific image processing, it is used as the subsequent classification network input.

### 2.4. Image Processing Algorithm

A specific image processing algorithm is designed to realize the smooth connection of the two networks for making the output image of the segmentation network better adapt to the classification network. At the same time, we also achieve the segmentation of each nodule in the multinodule image and complete the image classification of the nodule part.

Aiming at the situation that the pixel value of the output image of the segmentation network is a floating-point value, in order to facilitate the classification network learning, according to several attempts to verify, a threshold of 0.3 is designed to binarize the background and nodule of the output image.

The output of the segmentation network may have a small range of noise color blocks caused by some misjudgment. The length width ratio and the total area limit are designed to eliminate the interference without excluding the small-area nodules.

A single CT image will have multiple nodules. In order to avoid the degradation of classification effect caused by the hybridity of multiple nodules and single nodules, OpenCV is used to analyze the minimum connected domain of all mask images, find out all single nodules, and lay them in the black background of 256 ∗ 256 pixels according to the position of the original image, so as to facilitate the subsequent mapping to the original image for visualization. In addition, according to the pixel value of the corresponding mask image in the ROI region of the nodule, when the average pixel value tends to 1, the cut nodule is marked as benign nodule, and when the average pixel value tends to 2, it is marked as malignant nodule.

### 2.5. Image Classification Model

**Figure 3:**
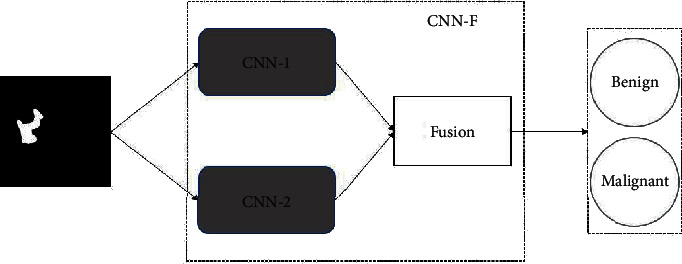
The basic structure of CNN-F.

The classification of benign and malignant thyroid nodules is based on CNN image classification [17]. The nodules of CT images are gray-black pixel blocks with subtle color differences, so the classification is mainly based on the shape and edge differences of benign and malignant nodules [18]. Based on this feature, a low-level and high-level feature fusion classification network CNN-F is designed. Its basic structure is shown in Figure 3. The shallow network is used to extract low-level image features, and the deep network is used to extract complex high-level image features. After fusion training, we can learn the performance of thyroid nodules at different characteristic levels.

**Figure 4:**
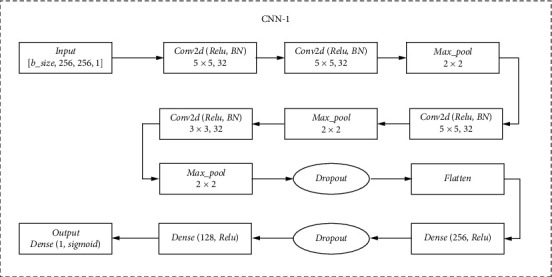
The architecture of CNN-1.

The shallow network CNN-1 is built independently, the main structure is shown in Figure 4. It is mainly composed of a combination of 3 parts of convolutional structure and pooling structure. The image features are extracted through the convolutional layer, and the resulting feature maps are compressed through the maximum pooling layer. On the one hand, the feature maps are reduced, and the network calculation complexity is simplified; on the one hand, feature compression is performed, the main features are extracted, and one dropout layer is added to avoid overfitting. Then, through the Flatten layer, the multidimensional input is made one-dimensional, and the transition from the convolutional layer to the fully connected layer is realized. Add two layers of fully connected layers and one layer of dropout, plus the nonlinear mapping of the activation function, to extract and integrate useful information to achieve the role of a classifier.

**Figure 5:**
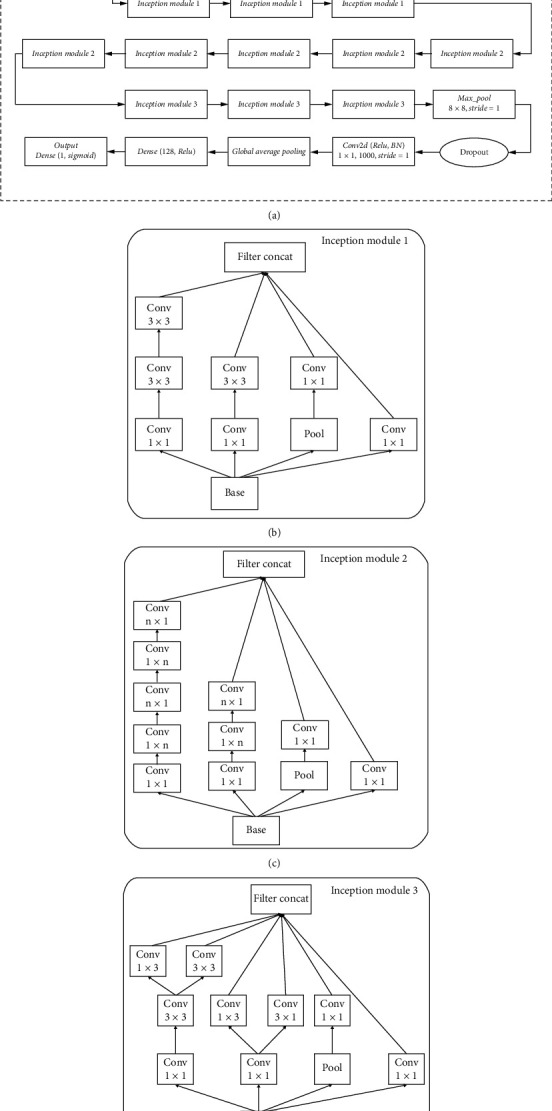
(a) The overall architecture of CNN-2. (b) The architecture of Inception module1. (c) The architecture of Inception module 2. (d) The architecture of Inception module 3.

The deep network structure CNN-2 uses the InceptionV3 [19] network as the basic structure, and the CNN-2 structure is shown in Figure 5. The Inception network structure is a “basic neuron” structure constructed by the GoogLeNet team to build a sparse, high-performance network structure. Convolution kernels of different sizes are used to enable the existence of different sizes of receptive fields. Finally, the splicing is achieved to achieve the fusion of features of different scales, which can not only maintain the sparsity of the network structure, but also utilize the high computational performance of the dense matrix. The InceptionV3 network introduces the idea of Factorization into small convolutions, which splits a larger two-dimensional convolution into two smaller one-dimensional convolutions. On the one hand, it saves a large number of parameters and reduces computing time and overfitting, while increasing a layer of nonlinear extended model expression capabilities [20]. The first module uses two 3 × 3 convolutions on a traditional basis instead of 5 × 5 convolutions. The second module reduces the feature map and adds filters and *n* × 1⟶1 × *n* structure, and the third module uses the convolutional pooling parallel structure. After calling the basic concept V3 (excluding the top-level classifier), the global average pooling layer [21] is added, which directly eliminates the black box feature in the full-connection layer, and gives each channel the actual internal meaning, greatly reducing the network parameters and avoiding overfitting. After that, a full-connection layer of 128 nodes is added to integrate the local feature map to get the global feature information.

After removing the output layer and using concatenate to splice the two models, 256 nodes are merged through the fully connected layer. Finally, the output layer uses the sigmoid activation function to predict the category and can also get approximate probability prediction, which can use probability to assist with classification decision [22].

### 2.6. Loss Function

In this experiment, binary dice loss is used to improve the loss of segmentation model. Loss is divided into Dice Loss and Binary Cross Entropy. Among them, the Dice coefficient is a measurement function used to measure the similarity of the set, which is actually used to calculate the pixels between samples [23]. The formula of Dice coefficient is as follows:

(1) $$\begin{array}{c} \begin{array}{c} s=\frac{2\left|X\cap Y\right|}{\left|X\right|+\left|Y\right|}\; \end{array}, \end{array}$$

where *X* represents the marked ground truth and *Y* represents the output image of the segmentation network. The formula of Dice Loss is defined as

(2) $$\begin{array}{c} \begin{array}{c} \text{loss}=1-\frac{2\left|X\cap Y\right|}{\left|X\right|+\left|Y\right|} \end{array}. \end{array}$$

Using Dice Loss as the loss function can achieve the real goal of segmentation more appropriately, optimize the evaluation criteria directly, and better deal with the imbalance of data. However, Dice Loss will have an adverse impact on the back propagation and easily make the training unstable [24]. Therefore, on the basis of Dice Loss, Binary Cross Entropy is added [25]. The calculation formula is as follows:

(3) $$\begin{array}{c} \text{Loss}=-\frac{1}{N}\overset{N}{\underset{i=1}{\sum }}y_{i}\;\cdot \;\log\;{\hat{y}}_{i}+\left(1-y_{i}\right)\;\cdot \;\log\left(1-{\hat{y}}_{i}\right), \end{array}$$

where *N* is the output size, *y*_*i*_ is the label, and $\;{\hat{y}}_{i}$ is the predicted probability of the label being positive for all *N* labels.

## 3. Results and Discussion

### 3.1. Experiment Setup

The experiment was conducted on a personal computer with NVIDIA GEFORCE RTX-2080Ti GPU and Intel^®^ Core™ i7-8700K Processor, 3.7 GHz. The experimental operating system is 64-bit Windows 10. The environment for deep learning is Python 3.6 and Keras 2.3.1 with TensorFlow GPU 1.14 as the back end. In the model training, the loss function used in segmentation network is Dice Loss + Binary Cross Entropy, the optimizer is Adam, and the performance evaluation index is IOU. In the classification network, CNN-1, CNN-2, and CNN-F used Binary Cross Entropy as the loss function, Adam as the optimizer, and ACC as the performance evaluation index. The initial learning rate of both methods was 0.001. The ReduceLROnPlateau callback function was used to dynamically adjust the learning rate. The attenuation coefficient was set to 0.5, and the cooldown was set to 10 epochs. A total of 200 epochs were trained. In addition, the parameter setting details of comparison methods are also consistent with the above, such as Vgg-16, DenseNet, and so on.

### 3.2. Performance Evaluation

The performance of the network model after concatenation can be evaluated by determining statistical values (recall, specificity, precision, and accuracy) and the *F*1-score. The recall reflects the positive proportion of correct recognition, the specificity reflects the negative proportion of correct recognition, the precision is the repeatability, or reproducibility of the measurement, and the accuracy is the proximity of measurement results to the true value. *F*1-score is an evaluation index which takes both precision and recall into account. The calculation formulas are shown as follows, where TP represents true positive, TN represents true negative, FP represents false positive, and FN represents false negative.

(4) $$\begin{array}{c} \begin{array}{c} \text{Accuracy}=\frac{\text{TP}+\text{TN}}{\text{TP}+\text{TN}+\text{FP}+\text{FN}}\;, \end{array} \end{array}$$

(5) $$\begin{array}{c} \begin{array}{c} \text{Recall}=\frac{\text{TP}}{\text{TP}+\text{FN}}\;, \end{array} \end{array}$$

(6) $$\begin{array}{c} \begin{array}{c} \text{Precision}=\frac{\text{TP}}{\text{TP}+\text{FP}}\;, \end{array} \end{array}$$

(7) $$\begin{array}{c} \begin{array}{c} \text{ Specificity}=\frac{\text{TN}}{\text{TN}+\text{FP}}\;, \end{array} \end{array}$$

(8) $$\begin{array}{c} \begin{array}{c} F1\text{ score}=2\cdot \frac{\text{Precision}\cdot \text{Sensitivity}}{\text{Precision}+\text{Sensitivity}} \end{array}. \end{array}$$

### 3.3. Semantic Segmentation Performances

**Figure 6:**
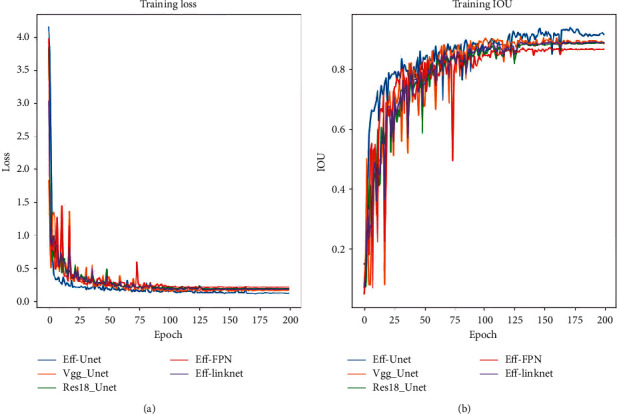
The training curve diagram of the training set.

In this experiment, in order to better segment the thyroid nodules and background parts, EfficientNetB4 was selected as the Unet model of the backbone network. In order to test the performance of the model, on the basis of Unet, we tried the common vgg backbone network and resnet18 backbone network. In addition, we also choose Linknet [26] and FPN [27] as the segmentation network and find that the performance of segmentation is worse than that of UNET in the case of EfficientNetB4 backbone network. The training curve diagram of the training set of various network structures is shown in Figure 6. The IOU scores of each method in the test set are shown in Table 3, in which Eff-Unet gets the highest score.

### 3.4. Classification Performances

In this experiment, the multiclass segmentation network of Unet structure was first tried. It aims to achieve three classifications of background, benign nodules, and malignant nodules at the segmentation level at one time. However, due to the nature of the image, the segmentation and classification effects are very poor. Therefore, under the condition of ensuring the performance of the segmentation network, in order to evaluate the CNN-F we designed, we have also implemented a variety of networks to extract nodule features for classification based on the morphology and edge features of the nodules. Among them, Vgg16 [28] and DenseNet [29] networks have been used by Jeong Hoon Lee in the classification of metastatic lymph nodes on CT images and have achieved excellent results [30]. The diagnostic performance of various network structures in the test data set is shown in Table 4. No matter in the comparison between Vgg16 and CNN-1, which is a shallow model, or in the comparison between DenseNet and CNN-2, which is a deep model, the single network model proposed in this experiment has advantages in performance. Moreover, the CNN-F obtained by fusing the shallow network and the deep network can clearly see that the performance is significantly improved compared to a single network model.

## 4. Discussion

**Figure 7:**
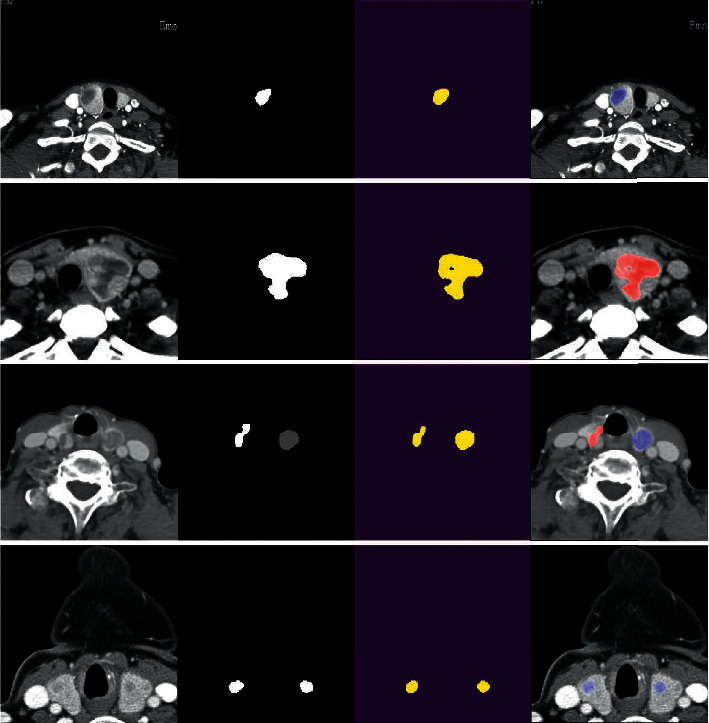
Visualization of test set results, in which the first column is the original image, the second column is manually labeled mask, the third column splits the nodule part of the network output, and the fourth column is the visualization result of the classification network output mapped to the original image. Blue represents benign nodules and red represents malignant nodules.

Timely diagnosis of benign and malignant thyroid nodules plays a key role in the treatment quality and prognosis of patients. Because there are complex tissues and organs in the CT images, unreasonable feature extraction and image preprocessing will lead to classification deviation, so machine learning algorithm cannot effectively solve such problems. While deep learning can improve the accuracy of classification or prediction by building a multihidden layer machine learning model and training with massive sample data [31]. This paper used a cascaded CNN method for intelligent recognition and classification of thyroid nodules with high accuracy without any annotation processing on CT images. When addressing the problem of imbalance in the number of benign and malignant nodules, class_weight was used, and the benign and malignant weight was set to 1 : 3 to balance the loss calculation. Test time enhancement [32] was used to further enhance the accuracy of the final result, predicting the original image, horizontal mirror image, vertical mirror image, and rotating image, and the final average value was taken as the final output. After effective segmentation of thyroid nodules by Eff-Unet, CNN networks with different depths were trained by fusion. Image features of different dimensions were extracted by the trained convolution filter and they were combined and normalized to achieve the classification of thyroid nodules.  Figure 7 shows the effect of some images of the test set in the system, in which the first column is the original image, the second column is manually labeled mask, the third column splits the nodule part of the network output, and the fourth column is the visualization result of the classification network output mapped to the original image. Blue represents benign nodules and red represents malignant nodules. The single nodule image can achieve clear classification, and the system can achieve accurate classification of each nodule for multiple nodule images classification.

## 5. Conclusion

In this work, we designed a set of end-to-end automatic recognition and classification system for thyroid nodules, which can realize the benign and malignant classification of nodules without any marking on CT images and can achieve accurate diagnosis of a single nodule, meeting the need of multinodule CT images. After comparing with the actual diagnosis report, compared with the common multiclass segmentation, we designed the segmentation network plus the classification network model, which greatly improves the accuracy of segmentation and classification. Finally, the total score of the algorithm is that the IOU score of the segmentation network is 0.856, and the overall classification performance has accuracy of 85.92%, recall of 91.43%, precision of 90.57%, specificity of 66.67%, ROC of 0.8253, and F1-score of 91.0% in this work. The overall performance of the system is shown in Table 5. The results show that the deep learning algorithm based on CNN can achieve accurate automatic diagnosis of thyroid nodules, provide doctors with reliable auxiliary report opinions, reduce the difficulty of doctors' work, and reduce the misdiagnosis rate. However, the current data is limited to enhanced CT images, and the database capacity is not large enough to prove the generalization of the model. Therefore, in the future research, we may focus on the relationship between ordinary CT and enhanced CT and further improve the model, trying to perfect the structure of the hybrid model to improve the robustness of the model as much as possible.

## Figures and Tables

**Table 1:** CT images distribution.

	Training and validation set	Test set	Total
Benign images	507	97	604
Malignant images	198	30	228
Total	705	127	832

**Table 2:** Thyroid nodules distribution.

	Training and validation set	Test set	Total
Benign nodes	543	103	646
Malignant nodes	243	34	277
Total	786	137	923

**Table 3:** The IOU scores of each method in the test set.

Method	IOU
Eff_FPN	0.768
Eff_Linknet	0.836
Res18-Unet	0.833
Vgg-Unet	0.842
Eff-Unet	**0.856**

**Table 4:** Classification performance results.

Method	Accuracy	Recall	Precision	Specificity	*F*1-score	ROC
Unet (3)	53.33	45.71	88.8	80.00	60.35	0.6394
Unet + Vgg16	71.11	75.49	87.50	63.33	81.05	0.7413
Unet + DenseNet	79.26	83.81	88.89	63.33	86.28	0.7704
Unet + CNN-1	80.74	86.67	88.35	60.00	86.51	0.7835
Unet + CNN-2	82.22	84.76	**91.75**	**73.33**	88.12	0.7968
Unet + CNN-F	**85.92**	**91.43**	90.57	66.67	**91.00**	**0.8253**

**Table 5:** The overall performance of the system.

IOU	Accuracy	Recall	Precision	Specificity	*F*1-score	ROC
0.856	0.859	0.914	0.906	0.667	0.910	0.825

## Data Availability

The image data used to support the findings of this study are available from the corresponding author upon request.
